# Enhancement of Virus Infection Using Dynamic Cell Culture in a Microchannel

**DOI:** 10.3390/mi9100482

**Published:** 2018-09-21

**Authors:** Jeong A Kim, Hye Jin Choi, Chul Min Kim, Hee Kyung Jin, Jae-sung Bae, Gyu Man Kim

**Affiliations:** 1 School of Mechanical Engineering, Kyungpook National University, 80 Daehakro, Buk-gu, Daegu 41566, Korea; kja588@naver.com (J.A.K.); hyejin0058@gmail.com (H.J.C.); faithfulsaint@hanmail.net (C.M.K.); 2 Osong Medical Innovation Foundation, 123 Osongsaengmyung-ro, Osong-eub, Heungdeok-gu, Cheongju-si, Chungbuk 28160, Korea; 3 Institute of Tissue Regeneration Engineering (ITREN), Dankook University, Cheonan 31116, Korea; 4 Department of Laboratory Animal Medicine, College of Veterinary Medicine, Kyungpook National University, 80 Daehakro, Buk-gu, Daegu 41566, Korea; hkjin@knu.ac.kr; 5 Department of Physiology, School of Medicine, Kyungpook National University, 680 Gukchaebosang-ro, Jung-Gu, Daegu 41944, Korea; jsbae@knu.ac.kr

**Keywords:** virus infection, microchannel, dynamic cell culture, induced pluripotent stem cells (iPSCs)

## Abstract

With increasing interest in induced pluripotent stem cells (iPSCs) in the field of stem cell research, highly efficient infection of somatic cells with virus factors is gaining importance. This paper presents a method of employing microfluidic devices for dynamic cell culture and virus infection in a microchannel. The closed space in the microchannel provided a better environment for viruses to diffuse and contact cell surfaces to infect cells. The microfluidic devices were fabricated by photolithography and soft lithography. NIH/3T3 fibroblast cells were cultured in the microfluidic device in static and dynamic conditions and compared with the conventional culture method of using Petri dishes. Virus infection was evaluated using an enhanced green fluorescent protein virus as a model. Dynamic culture in the microchannel showed similar growth of cells to that in Petri dish culture, but the virus infection efficiency was four-times higher. The proposed dynamic culture system could be useful in iPSC research by providing efficient virus infection tools.

## 1. Introduction

The first demonstration of the reprogramming of mouse fibroblasts into induced pluripotent stem cells (iPSCs) by Takahashi et al. in 2006 triggered a major paradigm change in the field of stem cell research [1]. Because iPSCs are derived from somatic cells and not from an embryo, they could solve an ethical problem in stem cell research, opening the possibilities of personalized stem cell therapy and disease models.

However, there are still several challenges that need to be addressed. One is that not all cells are reprogrammed during the reprogramming process. During the derivation process of iPSCs, somatic cells are infected with four factors, namely Oct4, SOX2, KLF4 and c-MYC [2,3]. The low efficiency of infection with virus factors results in very low reprogramming efficiency.

Recently, microtechnology and microfluidics have been applied increasingly in bioengineering and stem cell studies [4,5,6,7,8]. A microwell trapping device allowed one-to-one cell pairing based on cell fusion to improve reprogramming of somatic cells by embryonic stem cells [9]. Microstencils enabled localized culture of cells in microscale patterns to allow subsequent analysis in engineered manners [10,11]. Cell sorting, cell trapping, dynamic cell culture, embryoid body formation and virus infection in microfluidic devices have also been reported [12,13,14,15,16,17,18,19]. These applications of microtechnology offer control of stimuli and concentration gradients by taking advantage of hydrodynamic forces in the microscale channels.

Microfluidic devices can also be employed to improve reprogramming efficiency. Somatic cells are infected by specific virus factors, and the efficiency of viral infection is very important for the reprogramming of somatic cells into iPSCs. The conventional iPSC reprogramming process is conducted in Petri dishes. Virus factors are added to the culture medium and are floating until they are in contact with the somatic cell surface. Only a small number of virus factors floating around the cell surface could be used for infection, while most of them are discarded when the culture medium is replaced.

**Figure 1:**
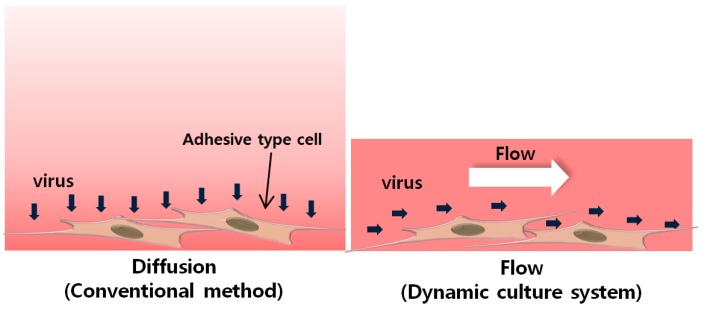
Cells subjected to virus infection by diffusion and flow.

The use of virus factors might be increased when microfluidic devices are employed. The main approach of this study for improving the efficiency is to increase the probability of contact of new viruses with the cell surface. Somatic cells are cultured in a microchannel while a mixture of culture medium and viruses flows through the microchannel (Figure 1). Because the flow continuously supplies viruses to the vicinity of the cell surface, the number of viruses in contact with cells is increased. At the same time, the number of viruses that are not used during the process will decrease, consequentially increasing the efficiency of virus infection.

In this study, a microfluidic device for dynamic cell culture was developed in order to enhance viral infection efficiency. Microchannel structures were fabricated by polydimethylsiloxane (PDMS) casting from microfabricated molds. NIH/3T3 cells were cultured in the microfluidic device in static and dynamic conditions. Cell viability and virus infection were evaluated and compared with those of conventional culture methods.

## 2. Materials and Methods

### 2.1. Microfluidic Device for Dynamic Cell Culture

**Figure 2:**
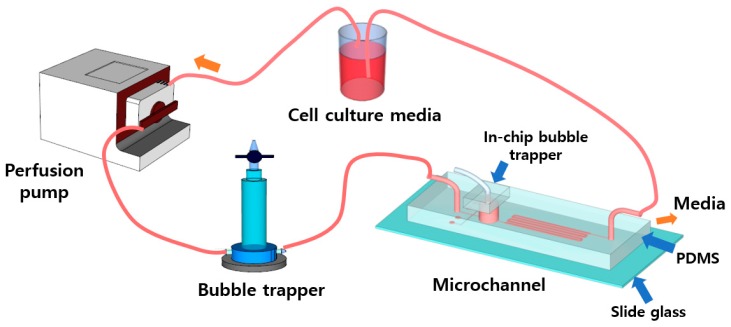
Dynamic cell culture system for virus infection.

As shown in Figure 2, the dynamic cell culture system is composed of a perfusion pump (ISMATEC, Wertheim, Germany), a bubble trapper, a microfluidic device and a culture medium reservoir. The devices are connected by a Tygon tube (Cole-Parmer, Vernon Hills, IL, USA) and placed in an incubator, except for the perfusion pump. The microchannel pattern of the microfluidic device was fabricated by casting from a mold that was fabricated by photolithography. Two types of molds, SU-8 and polyurethane acrylate (PUA), were used, depending on the height of the microchannel.

The perfusion pump was used to flow cell culture medium into the microchannel via the force of the rotating roll of the pump. The culture medium returns to the medium reservoir and circulates again into the cell culture system. A bubble trapper was used to avoid entrapment of bubbles inside the microchannel.

#### 2.1.1. Fabrication of the Microfluidic Device

The microfluidic device is composed of an in-chip bubble trapper (diameter: 5 mm), inlet/outlet channel and cell culture channel (width × length × height: 500 μm × 126 mm × 150 μm). The mold was fabricated by photolithography using SU-8 2100 photoresist (MicroChem, Westborough, MA, USA) on a silicon wafer. PDMS (Sylgard 184, Dow Corning, Midland, MI, USA) elastomer was replicated by casting from the fabricated mold. PDMS pre-polymer and curing agent were mixed at a 10:1 ratio and placed in a vacuum desiccator to remove bubbles. The mixture was poured onto the mold and cured in an oven at 90 °C for at least 2 h. After detaching the PDMS replica from the mold, to prevent microbubbles from forming in the microchannel, a bubble trapper hole was punched in the PDMS replica using a 5-mm biopsy punch [20]. Inlet and outlet holes were punched with a 1.5-mm biopsy punch, and the PDMS replica was placed on a slide glass to form a microchannel. Finally, the fabricated device was connected to the perfusion pump, culture medium reservoir and bubble trapper by Tygon tubes. Flow was driven by negative pressure to avoid leakage in the microchannel.

#### 2.1.2. Fabrication of an Ultra-Thick Microfluidic Device

**Figure 3:**
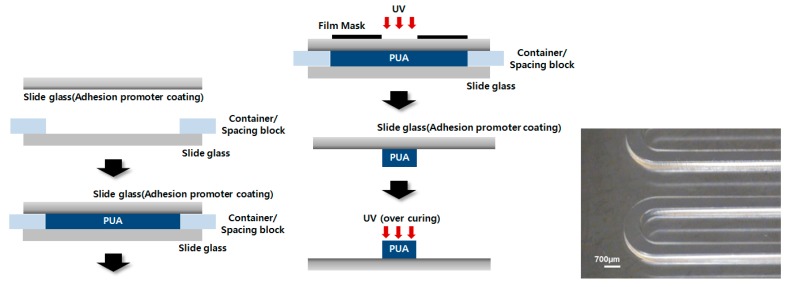
Schematic image of the PUA mold fabrication process and fabricated mold (thickness of 800 μm).

Although SU-8 is widely used to fabricate microstructures by photolithography, the height of the structures is limited. In order to investigate the effect of microchannel height from 300 μm–1000 μm, a new photo-polymer, PUA, was selected and an ultra-thick mold was fabricated, as shown in Figure 3. The resulting microchannel was 700 μm in width and 118 mm in length.

In order to promote adhesion of the cured micropattern with the slide glass, an adhesion promoter (Minuta, glass primer) was mixed with isopropyl alcohol (IPA, Duksan, Ansansi, Korea) at a 1:10 ratio. The adhesion promoter solution was coated on a slide glass by spinning at 3000 rpm. Two spacing blocks were placed on each end of the clean slide glass, and another slide glass and film-type photomask were placed on top of the clean slide glass [21]. The space between the two slide glasses was filled with PUA, which was polymerized by exposure to UV light. The thickness of the structure could be controlled according to the thickness of the spacing block. The polymerized PUA structures were rinsed using IPA and then exposed to weak UV light for 10 h. The PUA structures were used as a mold for microfluidic device preparation as described in Section 2.1.1.

### 2.2. Virus Infection in Cells

**Figure 4:**
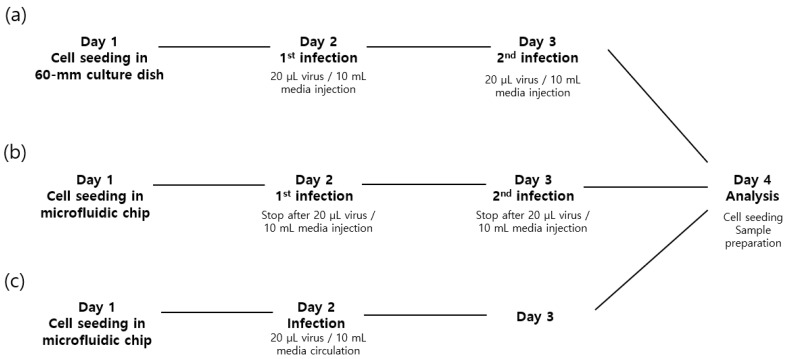
Schematic of experimental protocols of virus infection. (**a**) Conventional static culture (control); (**b**) static condition in the microchannel; (**c**) dynamic cell culture system.

NIH/3T3 cells were cultured and infected by viruses in three different conditions: conventional cell culture dish (control), static culture in a microchannel and dynamic culture in a microchannel. The schematic experimental protocols of virus infection are shown in Figure 4. Cell densities were set to be the same per unit area in all three culture conditions. Because all seeded cells should eventually settle on the bottom surface, the cell density per volume should be different to ensure the same initial cell density. Volume densities of 3 × 10^4^ cell/mL (dish) and 7 × 10^5^ cell/mL (microchannel) were used to provide an area cell density of 1 × 10^6^ cell/mm^2^.

In the conventional method, the NIH/3T3 cells were seeded on a disk in a 60-mm cell culture dish. The cells were grown in Dulbecco’s Modified Eagle’s Medium (Gibco, Grand Island, NY, USA) containing 10% fetal bovine serum (Gibco) and penicillin (100 units/mL penicillin-streptomycin, Gibco). After 1 day, the medium was replaced with 10 mL of medium supplemented with 20 μL of viruses every 24 h for 2 days. Enhanced green fluorescent protein (EGFP) was used as a model virus. On the next day, the cells on the disk were washed by phosphate-buffered saline.

In the case of static culture in the microchannel device, the microchannel was washed with 70% alcohol and deionized water and filled with medium using a 200-μL micropipette tip. NIH/3T3 cells in medium were filled in the bubble trapper and quickly pulled using a micropipette at the outlet of the microchannel in order to seed cells in the microchannel. The seeded cells were incubated at 37 °C in 5% CO_2_ for 1 day. The medium was replaced with 80 μL of medium mixed with 0.16 μL of virus. It took approximately 20 min to change the medium at a flow rate of 4 μL/min. The virus-mixed medium was replaced every 24 h for 2 days. On Day 4, the PDMS microchannel was carefully peeled off the slide glass, and the cells were inspected.

Lastly, in the dynamic cell culture system, the seeding condition was kept similar to that of the static culture method. The seeded cells were incubated for 4 h without any flow in the microchannel. Then, medium in a bottle was linked to the microchannel and circulated by the perfusion pump for 1 day. On the second day, EGFP viruses in medium were circulated for 2 days. On Day 4, the PDMS microchannel was carefully peeled off the slide glass, and the cells were inspected.

### 2.3. Fluorescence Analysis

Cell viability and virus infection were assessed by fluorescence imaging. For cell viability, the cells were cultured in the three different conditions described above. To assess the effect of culture environment in the microfluidic channel, no viruses were added. After 4 days of culture, the cells were dyed with the Live/Dead kit (Thermo Fisher Scientific, Waltham, MA, USA) and inspected under a fluorescence microscope (Olympus BX51, Tokyo, Japan). To obtain clear fluorescent images, the PDMS microfluidic devices were detached from the glass slide prior to inspection. The cell viability was calculated as the ratio of cell area over the total cell area. ImageJ was used to analyze the fluorescent images.

Virus infection tests were performed with the same procedure as that of the cell culture described in Section 2.2. From the fluorescent microscopic images, the areas of cells infected with EGFP were obtained. The virus infection efficiency was determined as a ratio of the area of infected cells over total cell area.

## 3. Results and Discussion

### 3.1. Effect of Microfluidic Culture System on Cell Viability

Compared to conventional cell culture in Petri dishes, microchannels could be harsh environments for cells because of the closed space and limited supplement of growth medium. Cell viability was observed in three different culture conditions: Petri dish (control) and microchannels with no flow (static) and flow (dynamic) of cell medium.

**Figure 5:**
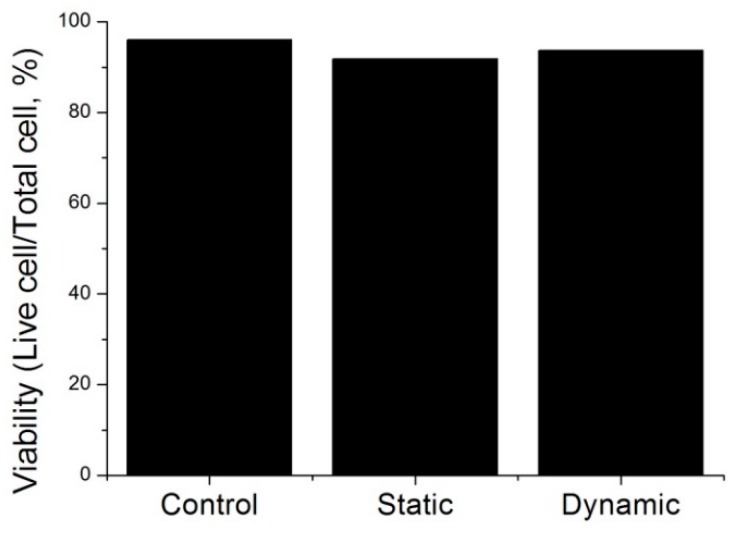
NIH/3T3 cell viability in three conditions. Sample size: *n* = 8; dimensions of the microchannel: 500 μm (W) × 126 mm (L) × 150 μm (H); flow rate of the dynamic culture: 4 μL/min.

Cell viability was evaluated following the cell culture protocol described in Section 2.2, but using culture medium instead of virus-mixed medium. Cell adhesion was observed 4 h after NIH/3T3 cell culture, and cell growth was observed after 4 days using a Live/Dead kit. The cells were inspected under a fluorescent microscope and eight fluorescent images taken at different positions. After confirming adhesion by cell culture with similar densities, the cell viabilities were shown to be similar at above 90% in all three conditions, as demonstrated in Figure 5, which indicates that the microchannels provided adequate conditions for cell culture.

### 3.2. Effect of Microfluidic Culture System on Virus Infection

Virus infection tests were performed following the experimental protocols described in Section 2.2, and the virus infection efficiency was examined at the three cell culture conditions. The dimensions of the microchannel were 500 μm (W) × 126 mm (L) × 150 μm (H). The flow rate of dynamic culture was 4 μL/min.

**Figure 6:**
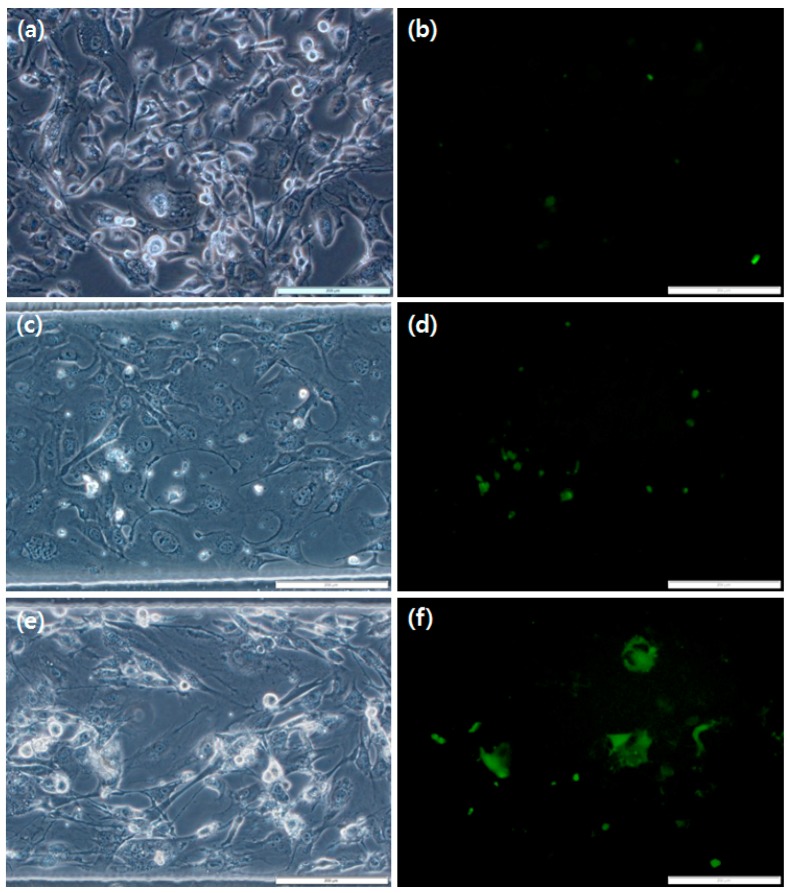
Optical images of NIH/3T3 cells and EGFP virus-infected cells after four days of culture. (**a**,**b**) Control, (**c**,**d**) static cell culture in microchannels and (**e**,**f**) dynamic cell culture system. Dimensions of the microchannel: 500 μm (W) × 126 mm (L) × 150 μm (H); flow rate of the dynamic culture: 4 μL/min, scale bar: 200 μm).

Figure 6 shows the optical images of cultured cells and fluorescent images of infected cells at the three culture conditions. The initial cell density was the same in all conditions at 1 × 10^6^ cell/mm^2^. On Day 4, the total cell areas were similar between the control and dynamic microchannel conditions, which were significantly larger than that in static microchannel condition. This is attributed to the reduced cell growth in static microchannel culture due to the limited supply of nutrients in the medium in the microfluidic channel.

**Figure 7:**
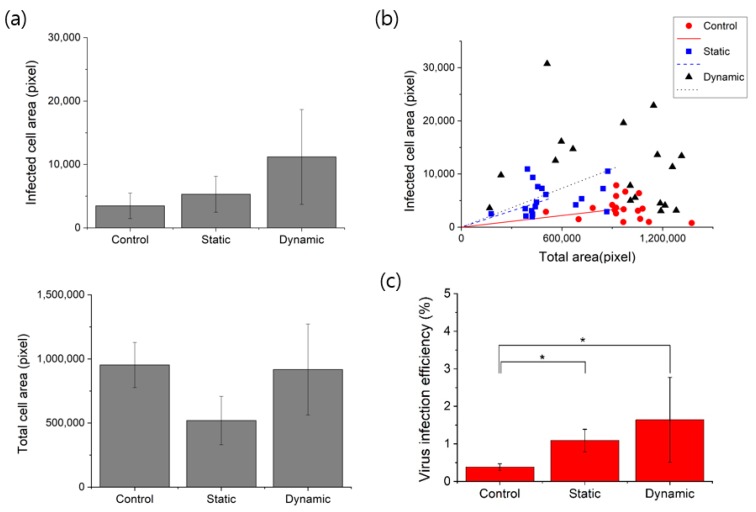
Virus infection in the conventional culture, static microchannel culture and dynamic cell culture system. (**a**) Comparison of virus-infected cell (EGFP) area and total cell area. (**b**) Comparison of virus infection efficiency (infected cell area against total cell area). (**c**) Efficiency of virus infection on NIH/3T3 cells. *t*-tests, * *p* < 0.01.

However, the infected cell area (EGFP) in the dynamic condition was significantly higher than those in the control and static condition, as shown in Figure 7a. At each condition, the virus infection test was repeated three times. Six fluorescent images were obtained at different positions along the microchannel. Figure 7b shows the infected cell area against total cell area from 18 fluorescent images of each condition.

The lines in Figure 7b indicate linear fitting of each condition, which shows the infection efficiencies. Comparing the static culture and the control, the infected cell areas were similar, whereas total cell area was higher in the control. Therefore, the infection efficiency of the static microfluidic culture was higher than that of the control. Dynamic culture showed better virus infection efficiency and cell growth than those of the static culture and higher infection efficiency than that of the control. With a flow rate of 4 μL/min, the dynamic culture system showed an increase in virus infection efficiency by 400% compared to that of the conventional method, as shown in Figure 7c.

### 3.3. Effect of Flow Rate and Virus Concentration on Virus Infection

**Figure 8:**
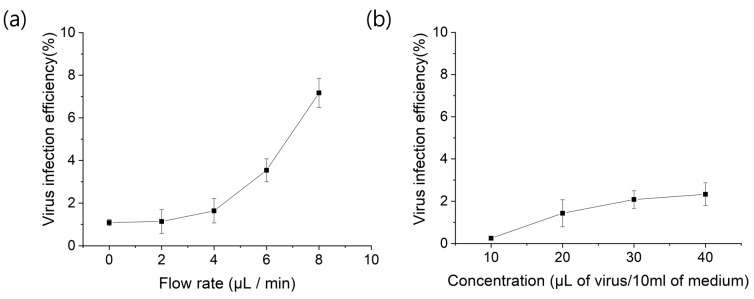
Virus infection efficiency of NIH/3T3 cells depending on (**a**) flow rate and (**b**) virus concentration. Dimensions of microchannel: 500 μm (W) × 126 mm (L) × 150 μm (H).

In dynamic microchannel culture, the flow rate can be regulated using a perfusion pump. Figure 8a shows the virus infection efficiency based on the flow rate of the virus-containing medium (concentration: 20 μL of virus/10 mL of medium). The dimensions of the microchannel were 500 μm (W) × 126 mm (L) × 150 μm (H). As the flow rate increased, the infection efficiency also increased. However, when the flow rate was greater than 6 μL/min, the cells began to detach from the glass slide’s surface.

Figure 8b shows the effect of virus concentration on infection efficiency. The flow rate was fixed at 4 μL/min, and 10–40 μL of viruses were mixed with 10 mL of culture medium. Higher virus concentrations showed higher efficiency. However, the efficiency was saturated at a concentration of higher than 30 μL of virus in 10 mL of culture medium.

### 3.4. Effect of Channel Height on Virus Infection

As observed in Figure 7a, the total cell area in the static culture was lower than that in the control and dynamic culture. This was attributed to a decline in cell growth in static microchannel culture due to the limited volume of nutrients in the medium within the microchannel (for example, 25-times smaller in unit area of the bottom surface in a 150-μm-thick microchannel than the control). Therefore, increased channel height may affect cell growth.

A PUA mold was used to fabricate microchannels of various heights. The fabrication of PUA molds is described in Section 2.1.2. Cover glasses were used as spacing blocks, and the thickness was adjusted by the number of cover glasses. Very thick mold structures with thicknesses of greater than 300 µm up to 1 mm were successfully fabricated. Microfluidic channels with channel heights of 300 µm–1 mm were then fabricated by PDMS casting. Cell viability and virus infection were examined following the cell culture protocol described in Section 2.2.

**Figure 9:**
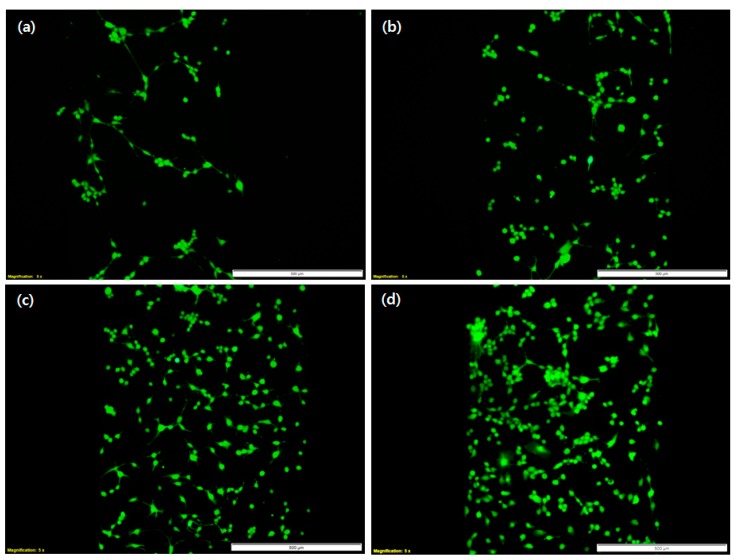
NIH/3T3 cell culture using the UV-curable plastic mold. Live (green) image after four days. Static culture with a channel height of (**a**) 150 µm, (**b**) 460 µm and (**c**) 800 µm and (**d**) dynamic culture with a channel height of 800 µm. Dimensions of the microchannel: 700 μm (W) × 118 mm (L); flow rate of the dynamic culture: 4 μL/min, Scale bar: 500 μm.

**Figure 10:**
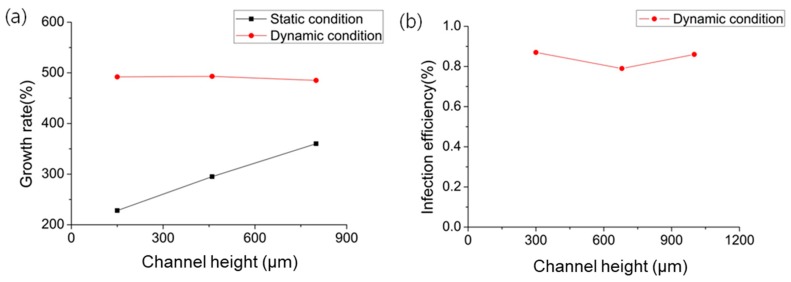
Growth rate of NIH/3T3 cells depending on the microchannel height. Dimensions of the microchannel: 700 μm (W) × 118 mm (L); flow rate of the dynamic culture: 4 μL/min.

Figure 9 shows the state of living cells in the microfluidic chip depending on the height of the microchannel and flow condition. Figure 9a–c shows the cell viability in static culture at microchannel heights of 150 µm, 460 µm and 800 µm. Figure 9d shows the cells in dynamic culture at a microchannel height of 800 µm. For the static culture condition, the number of live NIH/3T3 cells increased as the height of the microchannel increased, as shown in Figure 10a. On the other hand, dynamic cell culture maintained a high growth rate of cells, which remained constant regardless of the microchannel height. This is attributed to the continuous supply of nutrients by the dynamic cell culture system. Considering that the initial number of cells was similar in all conditions, the adequate supplement of medium and nutrients affects cell growth. Channel height did not exert significant effects on virus infection efficiency, as shown in Figure 10b, because the flow in the closed space of the microchannel was sufficient for viruses to spread efficiently onto the cell surface.

## 4. Conclusions

We presented a dynamic culture system for effective cell growth and virus infection. The dynamic culture system is composed of a perfusion pump, a bubble trapper, a microfluidic device and a culture medium reservoir. The microfluidic device was fabricated by photolithography and soft lithography. NIH/3T3 cells were cultured in the microfluidic device in static and dynamic conditions, and virus infection was evaluated using EGFP as a model virus. The static culture showed lower cell growth than that in the conventional Petri dish culture because of the limited supplement of culture medium, and cell growth was improved with increasing microchannel height. On the other hand, the dynamic culture showed similar cell growth to that in the Petri dish culture. Moreover, in dynamic culture, the virus infection efficiency was four-times higher than that in the Petri dish culture. The infection efficiency could be controlled by the flow rate and concentration of the virus-medium mixture.

The proposed dynamic culture system could be useful in iPSC research by providing efficient virus infection tools.
